# High‐Resolution IVIM MRI Characterizes Diffusion–Perfusion Properties of the Parasagittal Dura

**DOI:** 10.1002/nbm.70402

**Published:** 2026-09-18

**Authors:** Yi‐Jui Liu, Chun‐Han Liao, Shin‐Lei Peng, Shao‐Chieh Lin, Chao‐Chun Lin, Chia‐Wei Lin, Chia‐Hong Hsieh, Chun‐Wen Chen, Hing‐Chiu Chang

**Affiliations:** ^1^ Department of Automatic Control Engineering Feng Chia University Taichung Taiwan; ^2^ Ph.D. Program of Electrical and Communication Engineering Feng Chia University Taichung Taiwan; ^3^ Department of Medical Imaging Changhua Christian Medical Foundation Yuanlin Christian Hospital Changhua Taiwan; ^4^ Department of Medical Imaging Changhua Christian Hospital Changhua Taiwan; ^5^ Department of Biomedical Imaging and Radiological Science China Medical University Taichung Taiwan; ^6^ Department of Medical Imaging China Medical University Hospital Taichung Taiwan; ^7^ Department of Radiology Taichung Armed Forces General Hospital Taichung Taiwan; ^8^ Department of Medical Imaging and Radiological Sciences Central Taiwan University of Science and Technology Taichung Taiwan; ^9^ School of Medicine National Defense Medical University Taipei Taiwan; ^10^ Department of Biomedical Engineering The Chinese University of Hong Kong New Territories Hong Kong; ^11^ Multi‐Scale Medical Robotics Center New Territories Hong Kong

**Keywords:** DWI, IVIM, meningeal lymphatic vessels, parasagittal dura

## Abstract

The objectives of this study were to characterize the diffusion–perfusion properties of the parasagittal dura (PSD) using high‐resolution intravoxel incoherent motion (IVIM) MRI and to explore whether PSD‐specific IVIM metrics reflect distinct fluid‐transport‐related signal behavior compared with CSF and brain parenchyma. Ten healthy volunteers underwent 3 T MRI, including high‐resolution 3D T2‐FLAIR for anatomical localization and multi‐*b*‐value diffusion‐weighted imaging using MUSE and FOCUS IVIM sequences. Apparent diffusion coefficients (ADCs) and IVIM parameters—true diffusion coefficient (*D*), pseudo‐diffusion coefficient (D*), and perfusion fraction (*f*)—were quantified in the PSD, CSF, gray matter (GM), and white matter (WM). A pseudo‐diffusion residual analysis was performed to estimate the perfusion‐related *b*‐value transition. Tissue‐specific differences were assessed using nonparametric statistics. The PSD showed diffusion–perfusion characteristics distinct from those of both CSF and brain parenchyma. ADC values decreased with increasing *b* values and were intermediate between those of CSF and GM/WM. IVIM analysis demonstrated higher perfusion fractions and pseudo‐diffusion coefficients in the PSD than in GM and WM, indicating substantial microcirculatory or fluid‐transport contributions. The perfusion‐related *b*‐value transition thresholds in the PSD were higher than those in CSF but lower than those in GM and WM, supporting its intermediate perfusion behavior. MUSE and FOCUS acquisitions produced comparable quantitative metrics, with MUSE demonstrating reduced geometric distortion and FOCUS providing higher signal‐to‐noise ratios. In conclusion, the PSD exhibits a distinct diffusion–perfusion profile consistent with its role as a transitional interface among CSF pathways, dural lymphatics, and venous outflow. PSD‐specific IVIM metrics may provide a rapid, noncontrast approach for characterizing dural fluid‐transport‐related signal behavior at the interface between CSF spaces, meningeal lymphatics, and venous outflow.

## Graphical abstract

Parasagittal dura demonstrates intermediate diffusion and elevated perfusion‐related IVIM metrics compared with brain parenchyma, reflecting its unique microfluidic environment at the CSF–dura interface. IVIM imaging of the parasagittal dura provides a rapid, noncontrast MRI approach to assess dural fluid transport and may offer a potential imaging framework for evaluating brain fluid transport in neurological disorders.

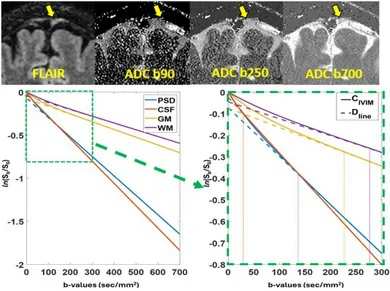

## 1 Introduction

Several fluid‐transport and clearance frameworks have been proposed to explain possible mechanisms of brain waste removal, including the glymphatic model [1], which proposes cerebrospinal fluid (CSF)–interstitial fluid (ISF) exchange through a combination of advection and diffusion, as well as alternative or complementary pathways such as intramural periarterial drainage (IPAD) and advection–diffusion “mixing” models of CSF–ISF transport [2]. Experimental studies, largely in animal models, together with emerging but indirect human imaging observations, suggest that alterations in brain fluid transport and waste‐clearance pathways may be associated with neurological disorders such as Alzheimer's disease, stroke, epilepsy, traumatic brain injury, and meningitis [3, 4]. However, the relationship between altered brain fluid clearance and neurological diseases in humans remains incompletely established and requires further validation in future studies. MRI techniques, including diffusion‐weighted imaging (DWI) and diffusion tensor imaging, have shown that apparent diffusion coefficient (ADC) measurements in perivascular or perivenous spaces may serve as potential biomarkers of impaired brain clearance [5, 6, 7].

The parasagittal dura (PSD) is a thin layer located on both sides of the superior sagittal sinus and along the brain convexities near the skull, with an approximate thickness of 1.14 ± 0.06 mm, and harbors abundant arachnoid granulations and lymphatic vessels [8]. However, its proximity to tissue–bone interfaces makes it highly susceptible to geometric distortion in conventional single‐shot echo‐planar DWI, leading to limited spatial resolution and frequent omission from diffusion studies. Our previous work demonstrated that water diffusion in the PSD can be quantified using high‐resolution Multiplexed Sensitivity Encoding (MUSE) and PROPELLER DWI, which provide reduced distortion and improved spatial resolution [7]. The results showed that PSD ADC values were found to lie between those of gray matter (GM), white matter (WM), and CSF, suggesting a distinct microenvironment and potential macromolecular accumulation.

Diffusion‐weighted MRI signals reflect both molecular diffusion and microcirculatory perfusion, and ADC derived from a mono‐exponential model cannot fully separate these effects. Intravoxel incoherent motion (IVIM) imaging enables simultaneous estimation of tissue diffusion (*D*), pseudo‐diffusion associated with microcirculatory flow (D*), and perfusion fraction (f) from DW‐MRI signal attenuation across multiple b values using a biexponential signal model [9, 10, 11].

The IVIM signal typically shows a “hockey‐stick” shape on logarithmic signal–*b* plots, with a rapid signal drop at low *b* values due to perfusion effects followed by a slower diffusion‐dominated decay at higher *b* values [12]. Because the perfusion effect is considered negligible at relatively high *b* values, a mono‐exponential function can be used in this high‐*b* range to estimate 𝐷, given an appropriate *b*‐value threshold [11, 13]. The threshold separating these regimes varies across tissues depending on microcirculatory characteristics [14, 15].

Although high‐resolution DWI has demonstrated the feasibility of measuring diffusion in the PSD [7], the diffusion and perfusion characteristics of this structure have not been systematically investigated. Therefore, this study aimed to characterize PSD diffusion–perfusion properties by evaluating ADC values across multiple *b* values, IVIM parameters, and the perfusion‐related *b*‐value transition using high‐resolution IVIM imaging.

## 2 Materials and Methods

### 2.1 Study Participants

Ten healthy volunteers (six males and four females; mean age, 30 years; range, 22–56 years) participated in this study (Figure S1). None had a history of neurological or cerebrovascular disease. The study protocol was approved by the Institutional Review Board of China Medical University Hospital (CMUH111‐REC1‐033), and written informed consent was obtained from all participants prior to enrollment.

### 2.2 MRI Data Acquisition

All MRI examinations were performed on a 3.0‐T system (SIGNA Architect, GE Healthcare) equipped with a dedicated head‐and‐neck coil. To reduce variability related to time‐of‐day effects on CSF and brain fluid dynamics, all MRI examinations were performed in the evening between approximately 19:00 and 22:00 [16]. The MRI protocol parameters are summarized in Table S1. To localize PSD, a high‐resolution three‐dimensional T2‐weighted fluid‐attenuated inversion recovery (T2‐FLAIR) sequence was first acquired. Subsequently, high‐resolution MUSE‐IVIM imaging with four‐shot acquisition and field‐of‐view optimized and constrained undistorted single‐shot (FOCUS) IVIM imaging with 30%–40% phase‐direction acquisition were acquired in sagittal and coronal planes oriented perpendicular to the PSD channel identified on the 3D FLAIR images. Finally, three‐dimensional T1‐weighted images were acquired using an MPRAGE sequence to provide anatomical reference for WM, GM, and CSF.

During image acquisition, high‐resolution 3D T2‐FLAIR images were used as the anatomical reference for PSD localization. On these images, the PSD appeared as a hyperintense band adjacent to the superior sagittal sinus. Multiplanar views in the coronal, sagittal, and axial planes were generated to guide DWI slice prescription. As shown in the supplementary figure (Figure S2), the orange dashed lines indicate the prescribed sagittal DWI slice positions, whereas the green dashed lines indicate the prescribed coronal DWI slice positions. These slices were positioned along the PSD region and oriented to include the thickest and most clearly visualized PSD segments, with the explicit aim of maximizing PSD coverage within the imaging slice while minimizing overlap with adjacent CSF spaces, cortical brain tissue, skull, and visible cortical or dural vessels. This acquisition strategy was designed to reduce contamination from neighboring structures as much as possible.

For motion‐artifact control, foam pads were used to stabilize the participant's head, and participants were instructed to maintain a stable head position throughout the MRI examination. After acquisition, images at each *b* value were carefully reviewed to evaluate inter–*b*‐value slice consistency and to identify motion‐related displacement, boundary blurring, ghosting, or morphological changes in the PSD and adjacent cortical structures. Datasets showing motion‐related displacement of the PSD, boundary blurring, ghosting, or mismatch of the PSD region across *b* values were excluded from the final analysis.

### 2.3 Data Processing and Quantitative Analysis

ADCs for each nonzero *b*‐value relative to *b* = 0 were calculated from the DWI images using the Stejskal–Tanner equation,

$$S_{b}/S_{0}=\exp\left(-\mathrm{bD}\right)\text{,}$$

where *S*₀ is the signal at *b* = *0* and *S*_*b*_ is the signal at each diffusion weighting (b > 0).

Diffusion data were analyzed using a bi‐exponential IVIM model, which separates the contributions of molecular diffusion and microcirculatory perfusion within each voxel according to the Le Bihan model:

$$S_{b}/S_{0}=f\times \exp\left(‐bD^{*}\right)+\left(1-f\right)\times \exp\left(-\mathrm{bD}\right)\text{,}$$

where *S*_*b*_ is the signal intensity at a given *b* value, *S₀* is the signal at *b* = *0*, *D* represents the true diffusion coefficient (reflecting pure molecular diffusion of water), D* is the pseudo‐diffusion coefficient associated with microvascular perfusion, and *f* denotes the perfusion fraction.

DWI signal fitting was performed using a two‐step approach to estimate the IVIM parameters. This segmented strategy has been shown to yield more robust estimates of *D*, D*, and *f* than single‐step global biexponential fitting when *b* values are not densely distributed, particularly in brain IVIM studies with limited scan time [13, 14, 17]. Because our high‐resolution protocol necessarily limited the number of *b* values that could be acquired, we adopted a segmented two‐step fitting approach to improve parameter stability under modest SNR and sparse *b*‐value sampling.

Based on previous brain IVIM studies, threshold *b* values of approximately 200–300 s/mm^2^ are commonly used to separate perfusion‐dominated and diffusion‐dominated signal components in brain parenchyma [14, 18]. In addition, recent CSF‐focused studies and theoretical modeling have shown that flow‐related pseudo‐diffusion effects are most prominent at low *b* values and become substantially smaller at higher diffusion weightings [19, 20]. Accordingly, we selected high‐*b* thresholds of ≥ 250 s/mm^2^ for the PSD, gray matter, and white matter, and ≥ 150 s/mm^2^ for CSF. Therefore, an initial diffusion coefficient *D* was obtained from a mono‐exponential fit at high *b* values (*b* ≥ 250 s/mm^2^; *b* ≥ 150 s/mm^2^ for CSF), and an initial perfusion fraction *f* was derived from the intercept of this diffusion component in the logarithmic signal–*b* plot [11]. Then, *D*, D*, and *f* were refined using constrained nonlinear biexponential fitting.

As shown in the supplementary figure (Figure S3), PSD ROI delineation was performed with reference to the corresponding high‐resolution 3D T2‐FLAIR images. First, the FLAIR slice closest to the planned DWI slice position was identified, and the PSD was recognized as a hyperintense band adjacent to the superior sagittal sinus. The corresponding PSD region was then manually delineated on the MUSE and FOCUS DWI images with reference to the FLAIR anatomy, as indicated by the red ROIs. During ROI placement, visible vessels, adjacent CSF spaces, skull interfaces, and areas with obvious boundary blurring or motion‐related misregistration were carefully avoided. GM, WM, and CSF ROIs were generated from tissue‐segmented masks derived from DWI coregistered to T1‐weighted images using Statistical Parametric Mapping (SPM12) [21]. To minimize partial‐volume effects at tissue boundaries, the GM, WM, and CSF masks were eroded by two pixels. All ROIs were verified by a board‐certified neuroradiologist with 14 years of experience.

ADC values at each *b* value and IVIM parameters (D, D*, and *f*) were extracted from each ROI in sagittal and coronal planes, and mean values were calculated for PSD, GM, WM, and CSF. Signal‐to‐noise ratio (SNR) was assessed using the dual‐acquisition subtraction method [22]. Detailed methods are provided in the Supporting Information.

### 2.4 Perfusion–Diffusion Transition Analysis

From the experimental data, ROI‐specific simulated IVIM curves in the logarithmic signal–*b*‐value domain were generated for PSD, GM, WM, and CSF using the biexponential IVIM model with the fitted parameters (D, D*, and *f*). The curves were sampled from *b* = 0 to 700 s/mm^2^ at 1 s/mm^2^ intervals. For each ROI‐specific simulated IVIM curve (*C*_IVIM_), tissue‐specific *D* values were estimated by mono‐exponential fitting at *b* ≥ 300 s/mm^2^, and the corresponding “pure diffusion” lines (*D*_line_) were simulated over *b* = 0–700 s/mm^2^ using this mono‐exponential model. To account for differences in diffusion magnitude among tissues, both curves were normalized to their values at *b* = 700 s/mm^2^, yielding norm_*C*_IVIM_ and norm_*D*_line_. The pseudo‐diffusion residual, defined as the percentage difference between norm_*C*_IVIM_ and norm_*D*_line_, was then calculated as a function of *b* value to identify the transition from perfusion‐influenced to diffusion‐dominated signal decay for each ROI and tissue.

### 2.5 Statistical Analysis

Statistical analyses were performed using MedCalc Statistical Software Version 22.026 (MedCalc Software Ltd., Ostend, Belgium). Nonparametric approaches were used due to small sample size and nonnormal distributions (Shapiro–Wilk tests). ADC values and IVIM parameters are presented as median with interquartile range (IQR, 25th–75th percentiles). Differences in ADC values and IVIM parameters among tissues (PSD, CSF, GM, and WM) were assessed using the Friedman test. When significant, pairwise comparisons were performed using Dunn's post hoc tests with Bonferroni correction. A *p* value < 0.05 was considered statistically significant.

For the perfusion–diffusion transition analysis, transition *b* values are reported as median [IQR] based on ROI‐level values derived from coronal and sagittal measurements. Exploratory acquisition‐wise comparisons between MUSE and FOCUS were performed using paired ROI‐level data available from the same participants using the Wilcoxon signed‐rank test.

## 3 Results

### 3.1 Participant Inclusion

Ten healthy volunteers were initially enrolled in this study. Because the PSD is a small and thin structure, it is particularly susceptible to displacement outside the imaging slice due to subject motion, and DWI is highly sensitive to motion artifacts. Consequently, datasets with excessive motion‐related image degradation were excluded. As a result, PSD measurements were successfully obtained in nine participants for the MUSE‐IVIM acquisition (six males and three females; mean age, 29.4 years; range, 22–56 years) and in eight participants for the FOCUS‐IVIM acquisition (five males and three females; mean age, 28.6 years; range, 22–56 years).

### 3.2 Image Quality

**FIGURE 1:**
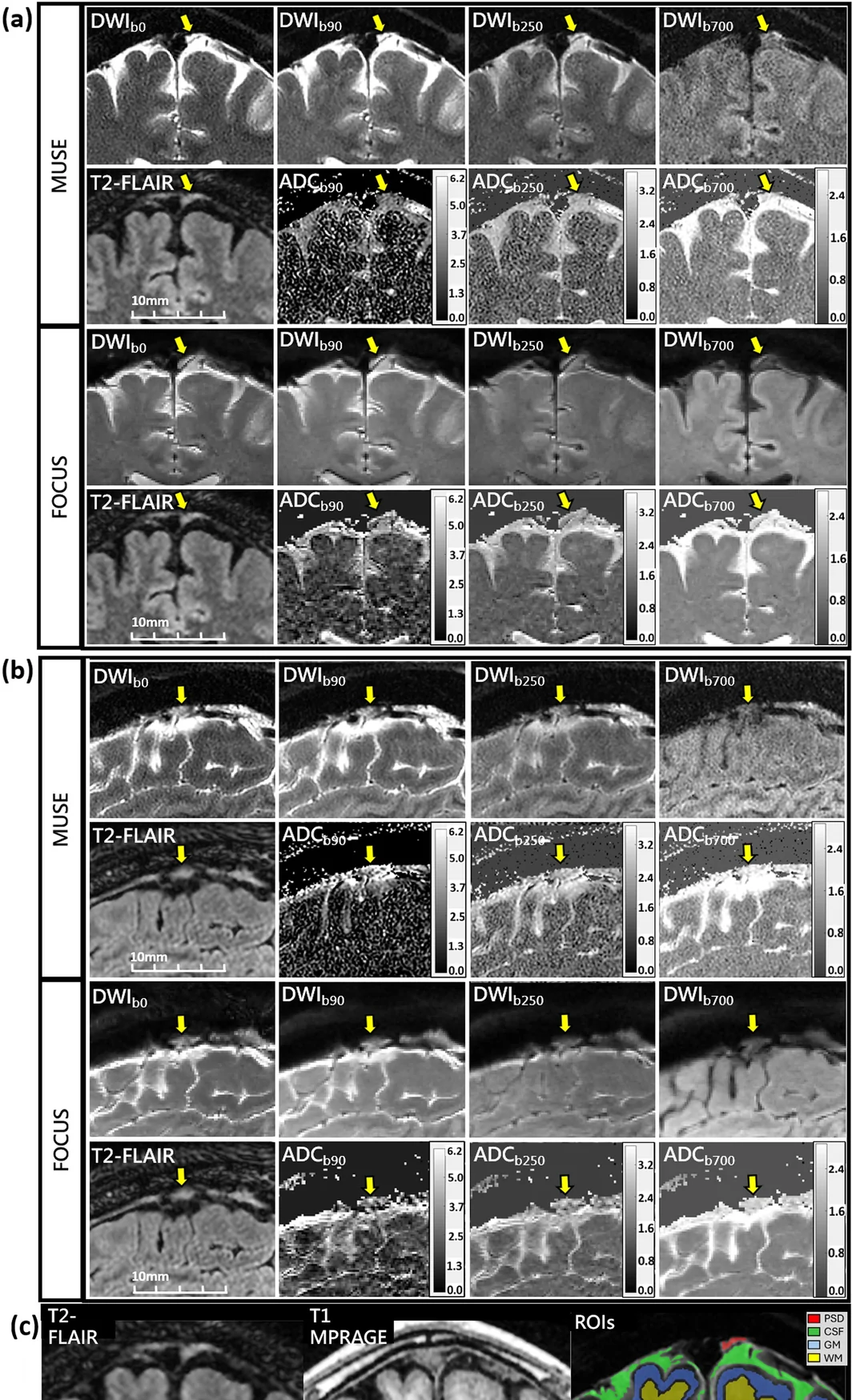
T2‐FLAIR, DWI (*b* = 0, 90, 250, and 700 s/mm^2^), and corresponding ADC maps acquired using MUSE and FOCUS methods in (a) coronal and (b) sagittal views. Spatial scale bars are shown in the FLAIR panels and also apply to the corresponding DWI and ADC panels within the same view. Intensity color bars are shown for the ADC maps, with ADC values expressed in ×10^−3^ mm^2^/s. The PSD (yellow arrows) becomes more clearly distinguishable from the adjacent CSF on higher *b*‐value ADC maps, particularly at *b* = 700 s/mm^2^, compared with lower *b* values ADC maps (*b* = 90 and 250 s/mm^2^). (c) T2‐FLAIR, T1‐weighted MPRAGE, and ROIs shown from left to right. ROI of PSD by manual draw and ROIs of CSF, GM, and WM using DWI coregistered to T1‐weighted images, the GM, WM, and CSF masks were eroded by two pixels to reduce the partial‐volume effects at tissue boundaries.

Figure 1 presents coronal and sagittal T2‐FLAIR, MUSE DWI, FOCUS DWI, corresponding ADC maps, and the ROIs for PSD, CSF, GM, and WM. Two additional representative cases are provided in the Supplementary Figures (Figure S4 and S5) in a format similar to Figure 1a,b, demonstrating that the PSD can be visualized across different subjects. Representative images at *b* = 90, 250, and 700 s/mm^2^ are shown, while the full *b*‐value range (30–700 s/mm^2^) is provided in the Supporting Information (Figure S6). MUSE also demonstrates reduced geometric distortion and improved PSD visualization compared with FOCUS, particularly at higher *b* values, while SNR was higher with FOCUS (Tables S2 and S3).

In our study, PSD ROIs were manually delineated on a single 2D slice. The in‐plane pixel size was 0.625 × 0.625 mm^2^ for MUSE and 0.755 × 0.784 mm^2^ for FOCUS. The PSD ROI area was 11.0 ± 6.0 mm^2^ for MUSE and 12.5 ± 6.4 mm^2^ for FOCUS (mean ± standard deviation across subjects), corresponding to approximately 28 ± 15 in‐plane pixels for MUSE and 21 ± 11 in‐plane pixels for FOCUS.

### 3.3 ADC Behavior Across *b*‐Values Pairs

Table 1 summarizes ADC values (median and IQR) obtained from MUSE and FOCUS DWI across eight *b*‐value pairs. ADC values decreased progressively with increasing *b* values, reflecting reduced perfusion contribution and greater sensitivity to true diffusion. Similar trends were observed for both MUSE and FOCUS acquisitions. The Friedman Test demonstrated significant differences in ADC among the four tissues, and subsequent pairwise comparisons showed that PSD ADC differed significantly from CSF, GM, and WM at all *b* values except at low *b* values (*b* = 10, 30, and 60 s/mm^2^) when compared with CSF, consistent with the observations in Figure 1.

**TABLE 1:** ADC values using MUSE and FOCUS for WM, GM, PSD, and CSF, which were calculated from eight pairs of *b* values (*b* = 0 paired with 10, 30, 60, 90, 150, 250, 500, and 700 s/mm^2^). Values are reported as median with interquartile range (P25–P75). The ADC value by *b* = 0 and *b* (10, 30, 60, 90, 150, 250, 500, and 700) is labeled as ADC_b_.

ADC ×10^−3^ mm^2^/s	PSD	CSF	GM	WM	Post hoc test
**MUSE**					
ADC_b10_	5.35 (4.16–6.46)	5.24 (4.82–5.62)	3.13 (2.44–3.72)	2.37 (1.36–2.60)	†
ADC_b30_	3.59 (3.36–4.17)	3.68 (3.52–3.84)	1.92 (1.65–2.24)	1.46 (1.10–1.69)	†
ADC_b60_	2.78 (2.45–3.50)	3.20 (3.03–3.27)	1.53 (1.31–1.73)	1.15 (1.05–1.24)	†
ADC_b90_	2.60 (2.42–2.83)	3.00 (2.89–3.14)	1.40 (1.24–1.56)	1.07 (0.94–1.14)	*
ADC_b150_	2.44 (2.17–2.70)	2.85 (2.78–2.98)	1.26 (1.16–1.34)	1.00 (0.96–1.05)	*
ADC_b250_	2.36 (2.18–2.65)	2.74 (2.70–2.87)	1.16 (1.08–1.21)	0.94 (0.91–0.96)	*
ADC_b500_	2.18 (2.06–2.41)	2.66 (2.52–2.75)	1.03 (1.01–1.09)	0.87 (0.85–0.90)	*
ADC_b700_	2.05 (1.99–2.35)	2.61 (2.45–2.68)	0.98 (0.96–1.02)	0.84 (0.82–0.87)	*
**FOCUS**					
ADC_b10_	5.13 (4.15–6.47)	5.48 (4.95–6.12)	3.08 (2.50–3.49)	1.26 (1.16–1.49)	†
ADC_b30_	3.73 (3.03–4.40)	3.52 (3.21–3.90)	1.87 (1.55–2.08)	1.11 (0.97–1.27)	†
ADC_b60_	3.24 (2.91–3.56)	3.09 (3.04–3.52)	1.52 (1.36–1.62)	0.94 (0.89–1.04)	†
ADC_b90_	2.89 (2.72–3.32)	3.09 (2.96–3.56)	1.40 (1.27–1.52)	0.96 (0.89–1.01)	†
ADC_b150_	2.71 (2.44–3.08)	2.90 (2.81–3.06)	1.26 (1.17–1.29)	0.93 (0.89–0.95)	*
ADC_b250_	2.48 (2.32–2.75)	2.78 (2.66–3.02)	1.15 (1.07–1.18)	0.88 (0.86–0.91)	*
ADC_b500_	2.43 (2.25–2.49)	2.66 (2.46–3.14)	1.01 (0.98–1.04)	0.84 (0.80–0.86)	*
ADC_b700_	2.23 (2.17–2.34)	2.53 (2.31–3.10)	0.95 (0.93–0.97)	0.80 (0.77–0.81)	*

* All pairwise comparisons among PSD, CSF, GM, and WM were significant (*p* < 0.05).

† PSD versus CSF: not significant (*p* ≥ 0.05); all other comparisons were significant (*p* < 0.05).

Figure 1 and Table 1 show that PSD and CSF had similar ADC values at low *b*‐value pairs (*b* = 10, 30, and 60 s/mm^2^ for MUSE; *b* = 10, 30, 60, and 90 s/mm^2^ for FOCUS), making them difficult to distinguish on ADC maps. At higher *b*‐value pairs, particularly *b* = 500–700 s/mm^2^, PSD ADC values were consistently lower than CSF ADC values, resulting in improved ADC‐map contrast and clearer visual delineation. This pattern is quantitatively supported by the post hoc comparisons in Table 1, where PSD–CSF differences were not significant at low *b*‐value pairs but became significant at higher *b*‐value pairs.

### 3.4 IVIM Parameter Profiles

**FIGURE 2:**
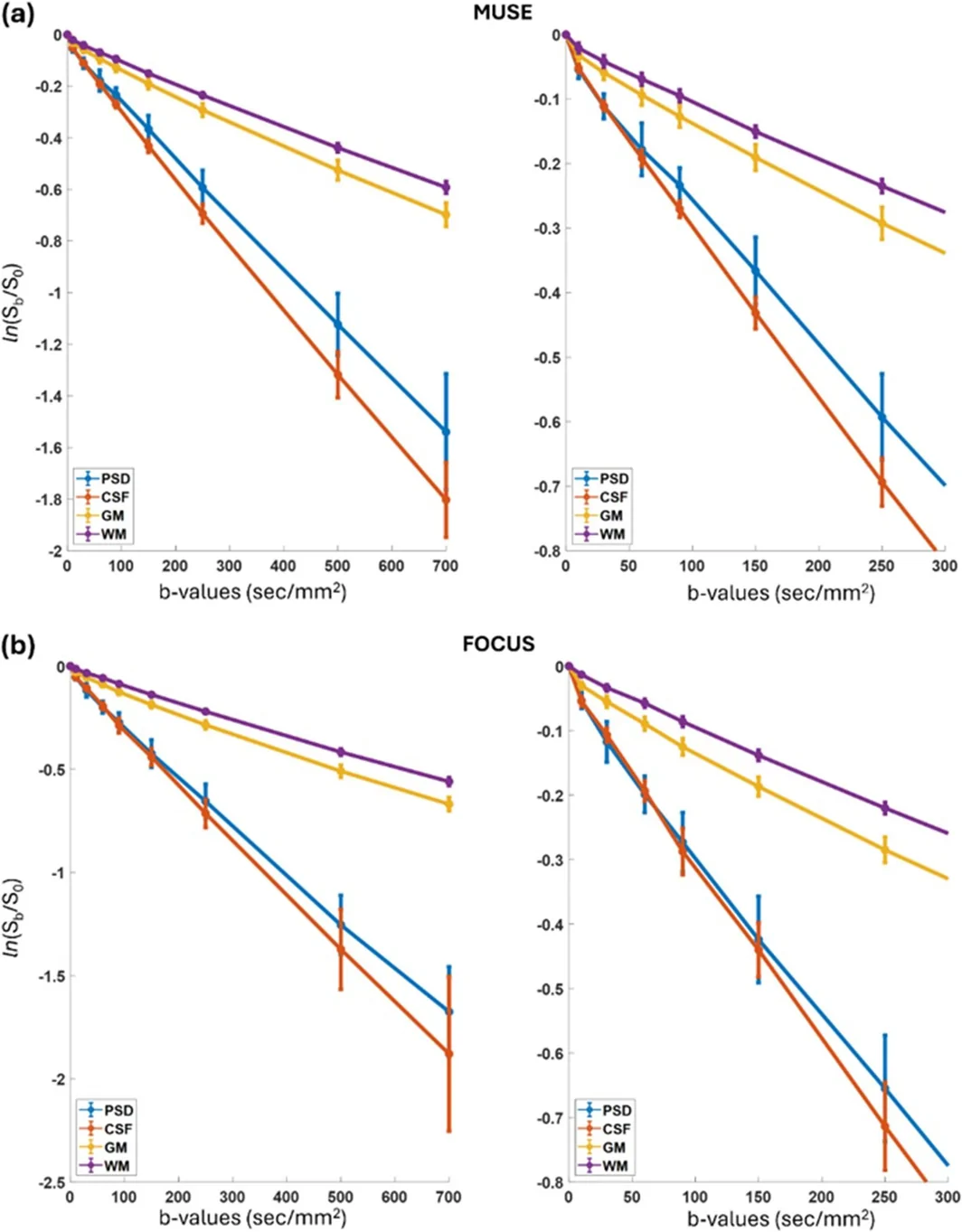
Semilogarithmic diffusion signal decay curves for PSD, CSF, GM, and WM obtained using (a) MUSE and (b) FOCUS DWI. The right panels show the full range of *b* values, while the left panels provide an enlarged view of the low *b*‐value range (*b* = 0–300 s/mm^2^) to highlight IVIM‐related curvature.

Figure 2 shows the DWI signal (mean ± standard deviation) of PSD, CSF, GM, and WM on semi‐logarithmic plots as a function of *b* value for both MUSE and FOCUS acquisitions. In both methods, signal decay followed an IVIM‐like pattern, with a steep decline at low *b* values and a more gradual decrease at higher *b* values. Signal‐decay patterns varied across tissues and depended on the *b*‐value range. PSD and CSF showed prominent and partly overlapping attenuation at low *b* values. At higher *b* values, particularly above approximately 90 s/mm^2^, the CSF and PSD curves became more clearly separated, whereas GM and WM showed more gradual signal attenuation across the examined *b*‐value range.

**FIGURE 3:**
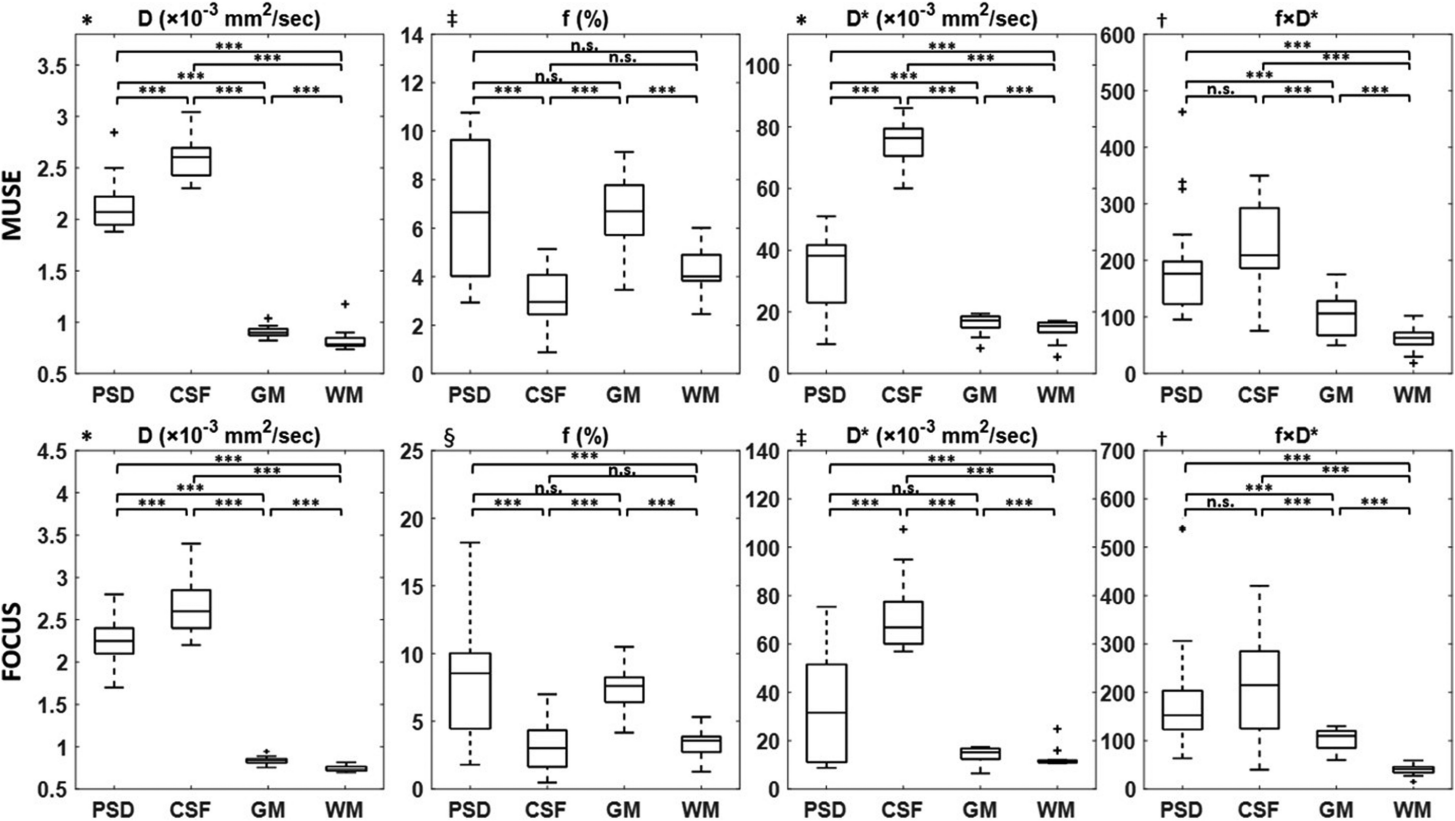
Boxplots of IVIM parameters (*D*, *f*, D*, and f·D*) in PSD, CSF, GM, and WM using MUSE (top) and FOCUS (bottom). Boxes indicate the interquartile range, horizontal lines indicate medians, whiskers indicate the nonoutlier range, and dots denote outliers. Pairwise post hoc comparisons are indicated by brackets; ***p < 0.001, and n.s. indicates *p* > 0.05. The symbols preceding each subplot title (*, †, ‡, and §) correspond to the statistical results reported in Table 2.

Table 2 summarizes the IVIM parameters (median, IQR) for the four tissues. MUSE and FOCUS acquisitions produced comparable parameter estimates and similar tissue‐dependent trends. The Friedman test showed significant differences among tissues for all IVIM parameters (*p* < 0.05). Post hoc Dunn–Bonferroni tests demonstrated tissue‐specific differences, with PSD generally differing from brain parenchyma, while some comparisons (e.g., PSD vs. CSF or PSD vs. GM) were not significant for certain parameters. Parameter distributions are illustrated in Figure 3.

**TABLE 2:** IVIM parameters (*D*, *f*, D*, and f·D*) for PSD, CSF, GM, and WM quantified using MUSE and FOCUS DWI are presented as median with interquartile range (P25–P75). Significant differences between tissues were assessed using Friedman test followed by Dunn's post hoc comparisons with Bonferroni correction (*p* < 0.05).

	PSD	CSF	GM	WM	Post hoc test
**MUSE**					
***D*** **×10** ^**−3**^ **mm** ^**2**^ **/s**	2.07 (1.95–2.22)	2.61 (2.43–2.69)	0.90 (0.87–0.93)	0.78 (0.77–0.85)	*
***f*** ***%***	6.66 (4.02–9.64)	2.95 (2.44–4.07)	6.69 (5.72–7.77)	4.01 (3.83–4.90)	§
D* **×10** ^**−3**^ **mm** ^**2**^ **/s**	38.19 (23.00–41.66)	76.33 (70.57–79.39)	17.22 (14.89–18.52)	15.42 (13.32–16.53)	*
f·D*	176.4 (123.1–198.2)	209.3 (186.3–292.8)	106.3 (67.5–128.3)	63.2 (51.6–72.5)	†
**FOCUS**					
***D*** **×10** ^**−3**^ **mm** ^**2**^ **/s**	2.23 (2.11–2.39)	2.58 (2.42–2.95)	0.85 (0.82–0.87)	0.75 (0.73–0.78)	*
***f*** ***%***	8.65 (6.00–10.88)	3.02 (1.62–4.41)	7.61 (6.29–8.41)	3.58 (2.69–3.87)	§
D* **×10** ^**−3**^ **mm** ^**2**^ **/s**	25.03 (11.22–41.97)	66.89 (60.00–78.39)	15.19 (12.31–16.77)	11.27 (10.90–11.89)	‡
f·D*	170.5 (124.4–307.2)	215.5 (119.5–298.2)	111.6 (81.4–118.3)	41.4 (32.5–46.1)	†

* All pairwise differences were significant (*p* < 0.001).

† PSD versus CSF: not significant (*p* ≥ 0.05); all other comparisons were significant (*p* < 0.001).

‡ PSD versus GM: not significant (*p* ≥ 0.05); all other comparisons were significant (*p* < 0.001).

§ PSD versus GM, CSF versus WM: not significant (*p* ≥ 0.05); all other comparisons were significant (*p* < 0.001).

### 3.5 Perfusion–Diffusion Transition Analysis

**FIGURE 4:**
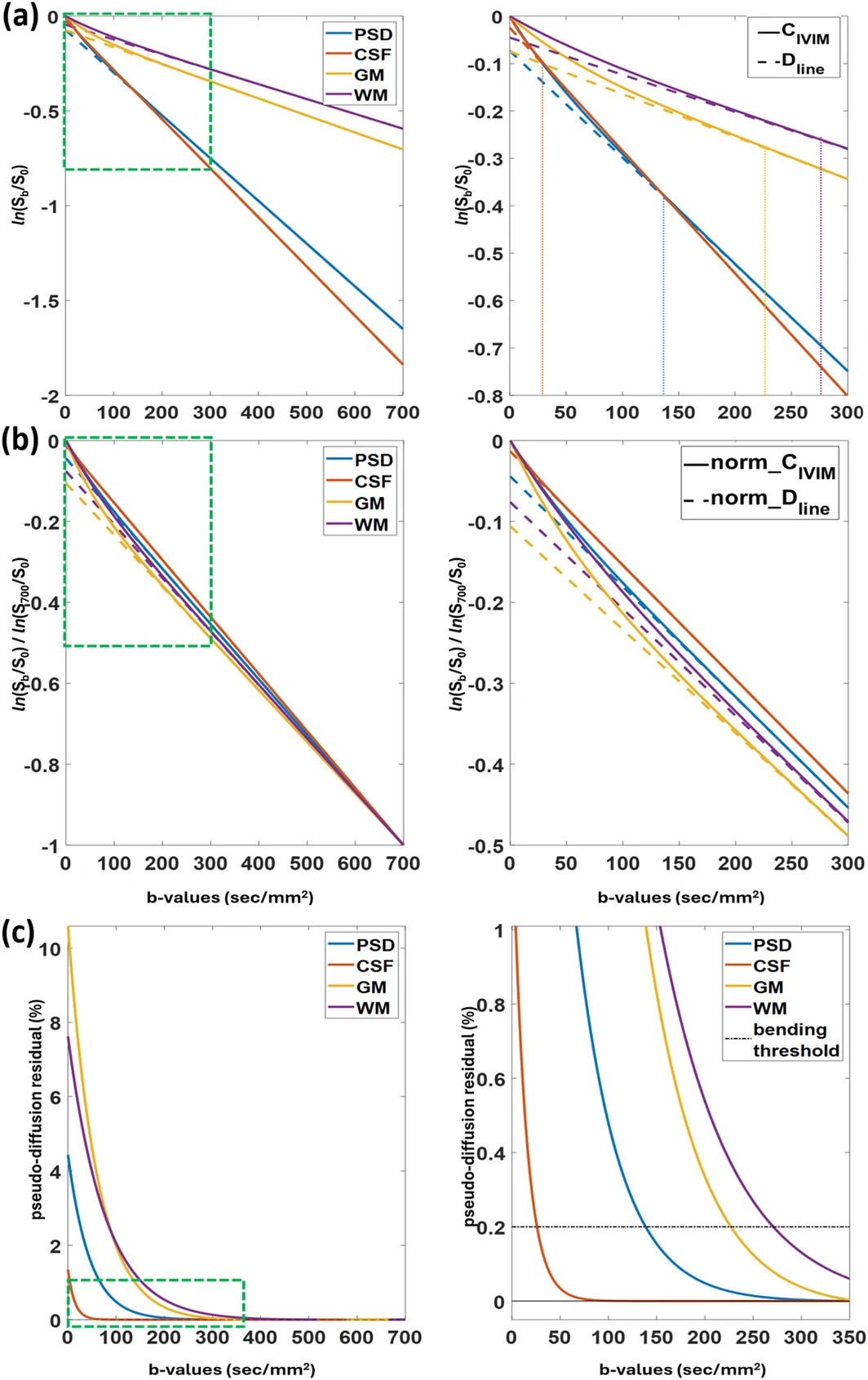
Representative subject‐specific simulated IVIM curves from the same participant with usable MUSE and FOCUS data, illustrating perfusion‐related signal behavior in the PSD, CSF, GM, and WM derived from MUSE data. (a) Semilogarithmic plots of the simulated IVIM signal (*C*_IVIM_) and diffusion line (*D*_line_). (b) Corresponding normalized curves (norm_*C*_IVIM_ and norm_ *D*_line_). (c) Percentage difference (pseudo‐diffusion residual) between norm_*C*_IVIM_ and norm_ *D*_line_ as a function of *b* value. In each row, the left panels show the full *b*‐value range, whereas the right panels provide an enlarged view of the low *b*‐value range highlighted by the dashed boxes. Vertical dotted lines in (a) show the *b* value at which the separation between *C*_IVIM_ and *D*_line_ becomes visually apparent, corresponding to the 0.2% pseudo‐diffusion residual threshold indicated by the horizontal dotted line in (c). The pseudo‐diffusion residual quantifies perfusion‐related signal contribution and identifies the transition toward diffusion‐dominated signal decay.

To assess perfusion effects in IVIM, simulated curves (*C*_IVIM_) were generated for PSD, CSF, GM, and WM by averaging subject‐specific simulations. Corresponding diffusion lines (*D*_line_) were derived and plotted in the logarithmic signal–*b* domain. MUSE‐based results are shown in Figure 4, while FOCUS results are provided in the Supporting Information (Figure S7).

Figure 4a,b show simulated IVIM curves and diffusion lines (*C*_IVIM_ and *D*_line_) and their normalized forms (norm_ *C*_IVIM_ and norm_ *D*_line_), together with enlarged view of curves highlighting the low *b*‐value region (0–300 s/mm^2^). Figure 4c presents the pseudo‐diffusion residual, defined as the percentage difference between the normalized IVIM curve and diffusion line (norm_*C*_IVIM_ − norm_ *D*_line_) as a function of *b* value, indicating the transition from perfusion‐influenced decay to mono‐exponential diffusion.

Transition *b* values are presented as median [IQR] in Table 3 for each tissue and residual threshold in both MUSE and FOCUS acquisitions. Exploratory acquisition‐wise comparisons between MUSE and FOCUS were performed using paired ROI‐level data, and no statistically significant differences in transition *b* values were detected for any tissue compartment at the evaluated residual thresholds (all *p* > 0.05). Across both acquisitions, CSF showed lower transition *b* values, whereas GM and WM showed higher transition *b* values. The PSD showed intermediate transition *b* values between CSF and brain parenchyma, supporting its intermediate perfusion–diffusion transition behavior.

**TABLE 3:** Transition *b*‐value thresholds (s/mm^2^) at specified pseudo‐diffusion residual levels for PSD, CSF, GM, and WM from MUSE and FOCUS IVIM analyses. Values are reported as median (IQR) based on ROI‐level transition *b* values. No statistically significant MUSE–FOCUS differences were detected for transition *b* values in any tissue compartment at the evaluated residual thresholds.

Pseudo‐diffusion residual (%)	MUSE, s/mm^2^, median (IQR)				FOCUS, s/mm^2^, median (IQR)			
	PSD	CSF	GM	WM	PSD	CSF	GM	WM
0.1	103.0 (89.0–173.5)	39.0 (35.0–46.5)	268.0 (259.0–285.0)	287.5 (266.5–344.0)	192.5 (93.0–311.5)	45.0 (28.0–57.0)	301.0 (283.0–311.0)	320.0 (284.5–384.0)
0.2	86.0 (70.5–143.0)	30.0 (26.0–36.5)	231.5 (223.0–249.5)	247.0 (229.0–299.0)	164.0 (75.5–277.0)	33.5 (18.5–45.0)	266.5 (244.0–278.5)	276.0 (243.0–340.5)
0.3	76.0 (59.5–125.0)	25.0 (20.5–31.0)	207.0 (201.0–226.5)	222.5 (206.5–271.0)	147.0 (66.0–251.5)	27.0 (14.0–38.0)	242.5 (218.0–255.5)	249.5 (218.5–313.0)
0.4	69.0 (52.0–112.5)	21.0 (17.5–27.0)	189.0 (184.0–209.5)	205.5 (189.5–251.5)	135.0 (59.5–231.0)	22.5 (9.5–33.0)	223.5 (198.5–237.0)	230.0 (201.5–291.5)
0.5	63.5 (46.5–102.5)	18.0 (14.5–23.5)	175.0 (170.5–195.5)	192.5 (176.5–235.5)	125.0 (55.0–213.5)	19.0 (7.0–29.0)	208.0 (181.5–222.0)	215.0 (187.5–275.0)

## 4 Discussion

### 4.1 MUSE and FOCUS Acquisitions

In this study, we used both MUSE and FOCUS IVIM sequences to characterize PSD diffusion–perfusion behavior. MUSE is a multishot EPI‐based DWI technique that corrects phase inconsistencies across shots, thereby yielding higher spatial resolution and reduced geometric distortion compared with conventional single‐shot EPI [23]. This makes it particularly advantageous for imaging thin structures near tissue–bone interfaces such as the PSD along the superior sagittal sinus. However, multishot acquisitions can be more sensitive to intershot motion and often require longer acquisition or reconstruction procedures. In contrast, FOCUS is a reduced field‐of‐view single‐shot EPI sequence that spatially constrains excitation to a limited region, thereby reducing susceptibility‐related distortion and shortening the EPI echo train [24]. These characteristics are highly beneficial for multi‐*b*‐value IVIM sampling within practical scan‐time constraints, though FOCUS provides more limited anatomical coverage and greater dependence on accurate slice prescription.

Consistent with these technical profiles, our empirical observations revealed that MUSE provided clearer anatomical delineation of the PSD with reduced distortion near the sinus wall, whereas FOCUS yielded higher SNR, which may facilitate more stable multi‐*b*‐value signal sampling. Despite these technical differences, PSD IVIM metrics, including ADC, D, f, D*, and f·D*, exhibited broadly similar qualitative patterns across both sequences. This consistency supports the feasibility of PSD diffusion–perfusion assessment when high‐resolution anatomical localization and careful ROI definition are applied. Given the pilot nature of this work, we interpret these sequence differences qualitatively and do not claim the superiority of one method over the other. Instead, MUSE and FOCUS should be viewed as complementary approaches: MUSE excels in geometric fidelity and precise PSD delineation, while FOCUS offers advantages in SNR and scan efficiency. Future studies incorporating larger cohorts, standardized protocols, and test–retest reliability assessments will be essential to determine the optimal acquisition strategy for PSD IVIM.

### 4.2 ADC With *b*‐Value Pairs Trends Across Tissues

ADC values derived from eight *b*‐value pairs showed tissue‐dependent diffusion behavior consistent with previous studies [25, 26]. With increasing *b* values, ADC decreased in the PSD, similar to GM and WM, reflecting reduced perfusion contribution and greater sensitivity to true molecular diffusion. PSD showed the highest ADC values at low *b* values, indicating greater microcirculatory and free‐water contributions, whereas GM and WM exhibited lower ADC values consistent with more restricted diffusion. These findings reflect tissue‐dependent diffusion–perfusion behavior and the sensitivity of ADC measurements to *b*‐value selection and microstructural properties.

### 4.3 IVIM Parameters Profiles of Dura, CSF, and Brain Parenchyma

For the true diffusion coefficient (*D*), CSF showed the highest values due to unrestricted water diffusion [27], while PSD exhibited intermediate values between CSF and brain parenchyma. GM and WM demonstrated lower *D* values, consistent with more restricted diffusion environments [11].

The pseudo‐diffusion coefficient (D*) was markedly higher in CSF than in GM and WM, indicating stronger perfusion‐related effects in fluid‐rich compartments [20, 28]. PSD also showed higher D* values than parenchymal tissues, suggesting greater fast incoherent motion related to microvascular or fluid transport, whereas GM and WM exhibited lower and more tightly distributed D* values.

These findings are consistent with recent IVIM–DTI and theoretical studies showing that cardiac‐driven and respiratory‐driven CSF pulsations, laminar and oscillatory flow in ventricular and subarachnoid spaces, and complex boundary conditions along perivascular and dural interfaces can generate prominent pseudo‐diffusion components at low *b* values [20, 28]. In IVIM terms, such heterogeneous CSF flow dynamics increase intravoxel velocity dispersion and thereby elevate D* in CSF compared with the more restricted, capillary‐dominated microcirculation in GM and WM. Therefore, the higher D* observed in CSF should be interpreted primarily as reflecting flow‐related incoherent motion and pulsatile CSF dynamics rather than tissue perfusion.

The perfusion fraction (*f*) is the fractional volume of water flowing in capillary compartment. In the study, it revealed marked tissue dependence, with the highest values in PSD, followed by GM, and lower values in CSF and WM. This pattern suggests a measurable perfusion‐ or fluid‐related signal contribution within the dural compartment, potentially reflecting microcirculatory or lymphatic flow.

The product f·D* as integration of perfusion‐related signal contribution can be as a metric to relative blood flow [10, 29]. The f·D* was highest in PSD and CSF, lower in GM, and lowest in WM, indicating stronger microcirculatory or fluid‐flow effects in dural and CSF spaces than in brain parenchyma.

In our study, we evaluated the perfusion‐related *b*‐value transition using the pseudo‐diffusion residual (norm_*C*_IVIM_ − norm_ *D*_line_) on the logarithmic signal–*b* plot to track where IVIM signal decay shifts from perfusion‐dominated to diffusion‐dominated behavior. This approach is consistent with the low‐ and high‐b separation in the original IVIM framework, with the characteristic “hockey‐stick” appearance of the IVIM curve [12], and with the threshold *b* values commonly used in segmented IVIM fitting [18, 30, 31]. Our results demonstrate that the transition *b* values in PSD differ from those in CSF and brain parenchyma, indicating a distinct perfusion‐related IVIM profile compared with well‐characterized brain tissues.

### 4.4 Anatomical and Fluidic Features Underlying PSD IVIM Behavior

The distinct IVIM profile observed in the PSD compared with CSF, GM, and WM may be related to its unique anatomical composition and fluid‐containing microenvironment. Anatomically, the PSD lies along the superior sagittal sinus and contains arachnoid granulations, intradural channels, and meningeal lymphatic vessels, forming a fluid‐containing stromal compartment that differs from the cellular architecture of GM and WM and from free CSF spaces [32, 33, 34, 35].

In vivo tracer studies have demonstrated CSF tracer passage or enrichment within the parasagittal dura and dural stroma, suggesting that the PSD may function as an interface linking CSF pathways, dural lymphatic structures, and venous sinuses rather than as a simple fibrous membrane [32, 33]. These fluid‐containing structures likely contribute to the relatively high perfusion fraction, pseudo‐diffusion coefficient, and f·D* observed in the PSD, supporting its distinct IVIM characteristics compared with brain parenchyma and CSF. However, the present data do not demonstrate greater CSF–tissue exchange at the PSD than at other CSF interfaces, and the mechanistic relationship between PSD IVIM metrics and CSF–dural exchange remains to be validated in future studies.

### 4.5 Potential Applications of PSD IVIM

Gadolinium‐enhanced MRI can characterize glymphatic and CSF–lymphatic transport by visualizing CSF–interstitial fluid exchange and downstream efflux pathways, including tracer propagation to the parasagittal dura and dural lymphatic channels [4]. These approaches have demonstrated impaired glymphatic transport in idiopathic normal pressure hydrocephalus, subarachnoid hemorrhage, acute ischemic stroke, and cerebral small vessel disease, as reflected by delayed clearance and prolonged tracer retention or enhancement [36, 37, 38, 39, 40]. Such findings suggest impaired drainage that can be quantified using dynamic T1‐weighted and 3D FLAIR imaging.

However, contrast‐enhanced protocols typically require repeated imaging over several hours to capture peak enhancement, and residual tracer is often evaluated at 24–48 h [4]. Although they provide valuable mechanistic insight, their invasive administration, gadolinium exposure, and prolonged imaging windows limit their suitability for longitudinal monitoring and routine clinical use.

IVIM enables quantitative assessment of diffusion and microcirculation without contrast administration and has been widely applied in liver fibrosis, oncology, stroke, cerebral small vessel disease, dementia, and insomnia [29, 41, 42, 43, 44]. Given the role of the parasagittal dura as a downstream interface for CSF and solute efflux toward dural lymphatic and venous outflow pathways, PSD‐specific IVIM metrics may provide a rapid, noncontrast approach for characterizing dural fluid‐transport‐related signal behavior at this interface. These metrics may serve as candidate imaging markers for glymphatic‐related drainage in future mechanistic and clinical research, particularly when combined with complementary structural and tracer‐based techniques. However, the current protocol has important limitations that preclude immediate clinical translation, including the 3‐mm slice thickness, which remains suboptimal for the thin PSD, limited SNR, and susceptibility to motion‐related artifacts during the relatively long IVIM acquisition. Therefore, PSD IVIM should be regarded as an exploratory approach for characterizing dural diffusion–perfusion behavior and hypothesized clearance pathways, rather than as a clinically viable diagnostic tool at this stage.

## 5 Limitations

This study characterized IVIM properties of the PSD, but several limitations should be noted. First, despite high‐resolution DWI, the 3‐mm slice thickness remains suboptimal for the thin PSD and may introduce partial‐volume effects from adjacent CSF, parenchyma, and cortical veins. Second, limited SNR required ROI‐based rather than voxelwise fitting for parameter estimation. Third, only nine *b* values were acquired (maximum 700 s/mm^2^), reflecting a compromise between sampling density, scan time, and SNR. Fourth, IVIM measurements in CSF and GM are known to be influenced by cardiac‐driven pulsations and CSF flow [27, 45]. Given that PSD IVIM metrics may be influenced by vascular and CSF pulsatility due to its proximity to venous sinuses and CSF spaces. Fifth, the small sample size and inclusion of healthy volunteers only limit generalizability.

Future work should incorporate higher resolution and SNR acquisitions, such as multishot 3D IVIM acquisitions [46], to improve the precision of PSD ADC and IVIM measurements. Larger cohorts and more extensive *b*‐value sampling are also needed to validate the reliability and sensitivity of PSD IVIM metrics. In addition, the influence of CSF pulsatility should be evaluated using approaches such as FLAIR‐DWI for CSF suppression and cardiac or pulse gating to reduce pulsatility‐related artifacts [47, 48].

Although scan timing was restricted to an evening time window, individual circadian phase, sleep status, and prior activity were not specifically controlled; therefore, residual circadian or physiological effects on PSD IVIM metrics cannot be excluded.

## 6 Conclusions

The parasagittal dura demonstrates diffusion–perfusion characteristics distinct from brain parenchyma and CSF. PSD‐specific IVIM metrics may provide a rapid, noncontrast approach for characterizing dural diffusion–perfusion behavior at the interface between CSF spaces, meningeal lymphatics, and venous outflow, but their relationship to CSF–dural exchange and glymphatic‐related drainage requires further validation.

## Author Contributions

Conceptualization: Yi‐Jui Liu, Chun‐Han Liao, Chun‐Wen Chen, Hing‐Chiu Chang. Investigation: Yi‐Jui Liu, Chun‐Han Liao. Methodology: Yi‐Jui Liu, Shin‐Lei Peng, Shao‐Chieh Lin. Data acquisition: Shin‐Lei Peng, Chao‐Chun Lin, Chia‐Wei Lin, Chia‐Hong Hsieh. Data analysis: Yi‐Jui Liu, Shao‐Chieh Lin. Funding acquisition: Yi‐Jui Liu, Chun‐Han Liao, Hing‐Chiu Chang. Writing – original draft: Yi‐Jui Liu. Writing – review and editing: Yi‐Jui Liu, Chun‐Han Liao, Chun‐Wen Chen, Hing‐Chiu Chang.

## Funding

Yi‐Jui Liu received financial support partly from the National Science and Technology Council of Taiwan (114‐2221‐E‐035‐036). Chun‐Wen Chen received financial support partly from the Taichung Armed Forces General Hospital (TCAFGH‐D‐114032). Hing‐Chiu Chang received financial support partly from the Hong Kong Research Grant Council (GRF14213225, GRF 17106820, GRF 17125321, and ECS 24213522).

## Supporting information




**Figure S1:** Flowchart of the study. Ten healthy volunteers underwent 3.0‐T MRI, including T2‐FLAIR, T1‐weighted imaging, MUSE‐DWI, and FOCUS‐DWI. Repeated scans were acquired in four participants for signal‐to‐noise ratio (SNR) estimation. ROIs were delineated in the parasagittal dura (PSD), cerebrospinal fluid (CSF), gray matter (GM), and white matter (WM). After exclusion of datasets with motion‐related artifacts or insufficient PSD visualization, PSD ROIs were successfully obtained in nine participants for MUSE‐DWI and eight participants for FOCUS‐DWI. Quantitative analyses included ADC estimation, SNR estimation, and intravoxel incoherent motion (IVIM) parameter calculation.
**Table S1:** The parameters of MRI protocols in the study.
**Figure S2:** Example of PSD‐targeted DWI slice prescription. High‐resolution 3D T2‐FLAIR images were used as the anatomical reference to identify the PSD as a hyperintense band adjacent to the superior sagittal sinus. Multiplanar reconstruction was used to prescribe DWI slices along the PSD region. The orange dashed lines indicate the prescribed sagittal DWI slice positions, and the green dashed lines indicate the prescribed coronal DWI slice positions. Slice locations were selected to include the thickest and most clearly visualized PSD segments while minimizing overlap with adjacent CSF, cortex, skull, and visible vessels.
**Figure S3:** Example of PSD ROI delineation. The upper row shows sagittal images from 3D T2‐FLAIR (left), MUSE IVIM DWI (middle), and FOCUS IVIM DWI (right), and the lower row presents enlarged views of the same slices. The PSD was identified on high‐resolution 3D T2‐FLAIR images as a hyperintense band adjacent to the superior sagittal sinus. The FLAIR slice closest to the DWI slice position was used as the anatomical reference, and the PSD ROI was manually delineated on the corresponding MUSE and FOCUS DWI images, as indicated by the red ROIs. Visible vessels, adjacent CSF spaces, skull interfaces, and areas with boundary blurring or motion‐related misregistration were avoided.
**Figure S4:** Additional case 1: T2‐FLAIR, DWI (b = 0, 90, 250, 700 s/mm2), and corresponding ADC maps acquired using MUSE and FOCUS methods in (a) coronal and (b) sagittal views.
**Figure S5:** Additional case 2: T2‐FLAIR, DWI (b = 0, 90, 250, 700 s/mm2), and corresponding ADC maps acquired using MUSE and FOCUS methods in (a) coronal and (b) sagittal views.
**Figure S6:** Representative coronal (a) and sagittal (b) images showing T2‐FLAIR, MUSE DWI, FOCUS DWI, and the corresponding ADC maps across multiple b values. For each plane, images acquired with MUSE are shown in the upper rows and those acquired with FOCUS in the lower rows. In each block, the first column displays the reference images (DWI b₀ in the upper row and T2‐FLAIR in the lower row). The subsequent columns show diffusion‐weighted images (upper row) and the corresponding ADC maps (lower row) for each b‐value pair (b = 0 paired with 30, 60, 90, 150, 250, 500, and 700 s/mm2). Yellow arrows indicate the parasagittal dura (PSD).
**Table S2:** DWI signal intensity obtained with MUSE and FOCUS for PSD, CSF, GM, and WM across all b‐values (0–700 s/mm2). Values are presented as mean ± standard deviation.
**Table S3:** SNR measured using the dual‐acquisition subtraction method for PSD, CSF, GM, and WM in MUSE and FOCUS across all b values (0–700 s/mm2). Values are mean ± standard deviation.
**Figure S7:** Representative subject‐specific simulated IVIM curves from the same participant with usable MUSE and FOCUS data, illustrating perfusion‐related signal behavior in the PSD, CSF, GM, and WM derived from FOCUS data. (a) Semilogarithmic plots of the simulated IVIM signal (CIVIM) and diffusion line (Dline). (b) Corresponding normalized curves (norm_CIVIM and norm_ Dline). (c) Percentage difference (pseudo‐diffusion residual) between norm_CIVIM and norm_ Dline as a function of b value. In each row, the left panels show the full b‐value range, whereas the right panels provide an enlarged view of the low b‐value range highlighted by the dashed boxes. Vertical dotted lines in (a) show the b value at which the separation between CIVIM and Dline becomes visually apparent, corresponding to the 0.2% pseudo‐diffusion residual threshold indicated by the horizontal dotted line in (c). The pseudo‐diffusion residual quantifies perfusion‐related signal contribution and identifies the transition toward diffusion‐dominated signal decay.

## Data Availability

The data are not publicly available due to privacy or ethical restrictions but may be obtained from the corresponding author upon reasonable request and with appropriate institutional approvals.
