# Supplementary material for: High‐Resolution IVIM MRI Characterizes Diffusion–Perfusion Properties of the Parasagittal Dura
Source: NMR Biomed. 2026 Sep 18;39(11):e70402. doi: 10.1002/nbm.70402 (PMC13586773; doi:10.1002/nbm.70402)
Supplement: Supplementary file 1 [file NBM-39-e70402-s001.docx]

Figure S1. Flowchart of the study. Ten healthy volunteers underwent 3.0-T MRI, including T2-FLAIR, T1-weighted imaging, MUSE-DWI, and FOCUS-DWI. Repeated scans were acquired in four participants for signal-to-noise ratio (SNR) estimation. ROIs were delineated in the parasagittal dura (PSD), cerebrospinal fluid (CSF), gray matter (GM), and white matter (WM). After exclusion of datasets with motion-related artifacts or insufficient PSD visualization, PSD ROIs were successfully obtained in nine participants for MUSE-DWI and eight participants for FOCUS-DWI. Quantitative analyses included ADC estimation, SNR estimation, and intravoxel incoherent motion (IVIM) parameter calculation.

Table S1. The parameters of MRI protocols in the study.

|  | **T2-FLAIR** | **T1 MPRAGE** | **MUSE** | **FOCUS** |
| --- | --- | --- | --- | --- |
| **TR (ms)** | 6000 | 2316 | 6000 | 4500 |
| **TE (ms)** | 146 | 2.1 | 90 | 74 |
| **TI (ms)** | 1700 | 1077 | NA | NA |
| **b-values (s/mm^2^)** | NA | NA | 0, 10, 30, 60, 90, 150, 250, 500, 700 | 0, 10, 30, 60, 90, 150, 250, 500, 700 |
| **NEX** | 1 | 1 | 1 | 2 (b=0), 1 (b=10, 30, 60, 90, 150, 250), 2 (b=500),  3 (b=700), |
| **FOV (mm)** | 200 × 200 | 200 × 200 | 200 × 200 | 200 × 80 |
| **Matrix Size** | 200 × 200 | 200 × 200 | 320 × 320 | 265 × 102 |
| **Slice thickness (mm)** | 1 | 1 | 3 | 3 |
| **Scan time (sec)** | 240 | 240 | 720 | 185 |

MPRAGE : Magnetization-prepared rapid gradient-echo

Figure S2. Example of PSD-targeted DWI slice prescription. High-resolution 3D T2-FLAIR images were used as the anatomical reference to identify the PSD as a hyperintense band adjacent to the superior sagittal sinus. Multiplanar reconstruction was used to prescribe DWI slices along the PSD region. The orange dashed lines indicate the prescribed sagittal DWI slice positions, and the green dashed lines indicate the prescribed coronal DWI slice positions. Slice locations were selected to include the thickest and most clearly visualized PSD segments while minimizing overlap with adjacent CSF, cortex, skull, and visible vessels.

Figure S3. Example of PSD ROI delineation. The upper row shows sagittal images from 3D T2‑FLAIR (left), MUSE IVIM DWI (middle), and FOCUS IVIM DWI (right), and the lower row presents enlarged views of the same slices. The PSD was identified on high-resolution 3D T2-FLAIR images as a hyperintense band adjacent to the superior sagittal sinus. The FLAIR slice closest to the DWI slice position was used as the anatomical reference, and the PSD ROI was manually delineated on the corresponding MUSE and FOCUS DWI images, as indicated by the red ROIs. Visible vessels, adjacent CSF spaces, skull interfaces, and areas with boundary blurring or motion-related misregistration were avoided.

Figure S4. Additional case 1: T2-FLAIR, DWI (b = 0, 90, 250, 700 s/mm²), and corresponding ADC maps acquired using MUSE and FOCUS methods in (a) coronal and (b) sagittal views.

Figure S5. Additional case 2: T2-FLAIR, DWI (b = 0, 90, 250, 700 s/mm²), and corresponding ADC maps acquired using MUSE and FOCUS methods in (a) coronal and (b) sagittal views.

Figure S6. Representative coronal (a) and sagittal (b) images showing T2-FLAIR, MUSE DWI, FOCUS DWI, and the corresponding ADC maps across multiple b-values. For each plane, images acquired with MUSE are shown in the upper rows and those acquired with FOCUS in the lower rows. In each block, the first column displays the reference images (DWI b₀ in the upper row and T2-FLAIR in the lower row). The subsequent columns show diffusion-weighted images (upper row) and the corresponding ADC maps (lower row) for each b-value pair (b = 0 paired with 30, 60, 90, 150, 250, 500, and 700 s/mm²). Yellow arrows indicate the parasagittal dura (PSD).

**Signal-to-noise ratio (SNR)**

Four participants were recalled for dual MRI acquisition under the same MRI scanner and protocols used in the main study. Each participant underwent two consecutive measurements for MUSE DWI and FOCUS DWI. The SNR was calculated using the dual acquisition subtraction method with the following formula:

SNR = $\sqrt{2} \mu_{signal}/\sigma_{diff}$

Where: 𝜇_signal_ is mean intensity of the signal region (ROI) from the first acquisition, and 𝜎_diff_ is the standard deviation of the corresponding ROI in the difference image (obtained by subtracting the two acquisitions).

ROIs for the PSD were manually drawn based on corresponding anatomical landmarks identified on 3D FLAIR images, with care taken to avoid visible vessels and partial-volume contamination. ROIs for GM, WM, and CSF were generated from tissue-segmented masks derived from DWI coregistered to T1-weighted images using Statistical Parametric Mapping (SPM12). Signal intensity across all b-values for PSD, CSF, GM, and WM measured using MUSE and FOCUS DWI, along with SNR analysis performed using the dual-acquisition (subtraction) technique, are provided in the Supplementary Information (Tables S1 and S2).

Table S2. DWI signal intensity obtained with MUSE and FOCUS for PSD, CSF, GM, and WM across all b-values (0–700 s/mm²). Values are presented as mean ± standard deviation.

| **Intensity** | PSD | CSF | GM | WM |
| --- | --- | --- | --- | --- |
| **MUSE** |  |  |  |  |
| DWI_b0_ | 6612.06 ± 2601.46 | 6972.23 ± 2054.41 | 3130.24 ± 776.26 | 2541.21 ± 611.76 |
| DWI_b10_ | 6270.92 ± 2488.04 | 6619.74 ± 1952.09 | 3030.94 ± 748.80 | 2487.79 ± 595.91 |
| DWI_b30_ | 5930.94 ± 2370.41 | 6243.08 ± 1840.14 | 2949.76 ± 724.98 | 2436.78 ± 583.54 |
| DWI_b60_ | 5544.51 ± 2202.02 | 5753.22 ± 1680.89 | 2847.45 ± 694.10 | 2371.79 ± 570.43 |
| DWI_b90_ | 5220.68 ± 2006.05 | 5313.15 ± 1547.63 | 2753.95 ± 670.36 | 2311.17 ± 554.90 |
| DWI_b150_ | 4570.01 ± 1735.67 | 4514.67 ± 1296.38 | 2584.08 ± 626.30 | 2185.59 ± 522.99 |
| DWI_b250_ | 3630.19 ± 1338.54 | 3468.13 ± 979.79 | 2332.72 ± 560.69 | 2008.92 ± 481.30 |
| DWI_b500_ | 2129.04 ± 760.89 | 1846.38 ± 491.50 | 1846.97 ± 440.36 | 1638.24 ± 390.63 |
| DWI_b700_ | 1386.05 ± 454.72 | 1132.92 ± 290.49 | 1554.27 ± 370.29 | 1405.56 ± 336.57 |
| **FOCUS** |  |  |  |  |
| DWI_b0_ | 5187.14 ± 2346.67 | 5763.84 ± 1672.25 | 3400.81 ± 939.62 | 2819.25 ± 731.96 |
| DWI_b10_ | 4913.72 ± 2210.09 | 5458.68 ± 1585.56 | 3299.39 ± 910.84 | 2785.05 ± 724.14 |
| DWI_b30_ | 4626.58 ± 2110.53 | 5177.60 ± 1495.17 | 3219.11 ± 893.65 | 2729.95 ± 709.72 |
| DWI_b60_ | 4272.86 ± 1930.83 | 4749.26 ± 1378.29 | 3110.05 ± 859.16 | 2666.13 ± 693.42 |
| DWI_b90_ | 3969.50 ± 1801.74 | 4321.49 ± 1245.64 | 3000.38 ± 828.26 | 2589.23 ± 672.38 |
| DWI_b150_ | 3450.65 ± 1622.10 | 3709.19 ± 1064.79 | 2821.46 ± 779.62 | 2457.35 ± 638.83 |
| DWI_b250_ | 2770.13 ± 1271.05 | 2823.23 ± 821.38 | 2557.05 ± 704.48 | 2262.99 ± 589.23 |
| DWI_b500_ | 1589.66 ± 754.89 | 1483.33 ± 496.44 | 2042.50 ± 559.45 | 1865.10 ± 483.83 |
| DWI_b700_ | 1096.23 ± 506.51 | 930.44 ± 384.82 | 1740.12 ± 474.10 | 1617.21 ± 420.81 |

Table S3. SNR measured using the dual-acquisition subtraction method for PSD, CSF, GM, and WM in MUSE and FOCUS across all b-values (0–700 s/mm²). Values are mean ± standard deviation.

| **SNR** | PSD | CSF | GM | WM |
| --- | --- | --- | --- | --- |
| **MUSE** |  |  |  |  |
| DWI_b0_ | 9.9±4.8 | 9.9±4.2 | 8.9±3.9 | 7.0±0.6 |
| DWI_b10_ | 15.4±8.7 | 12.7±6.4 | 13.4±8.2 | 10.6±1.8 |
| DWI_b30_ | 13.3±8.0 | 13.0±6.2 | 12.8±6.7 | 12.1±2.3 |
| DWI_b60_ | 17.0±10.1 | 12.8±5.7 | 13.4±6.6 | 11.1±1.2 |
| DWI_b90_ | 13.0±7.6 | 11.8±4.4 | 12.7±4.9 | 10.3±1.9 |
| DWI_b150_ | 13.5±7.4 | 11.0±3.7 | 12.7±4.6 | 10.8±2.2 |
| DWI_b250_ | 12.1±4.4 | 10.0±2.9 | 11.0±2.7 | 10.3±1.7 |
| DWI_b500_ | 8.4±3.2 | 7.9±1.7 | 9.4±1.1 | 8.7±1.1 |
| DWI_b700_ | 6.7±2.3 | 5.8±0.9 | 7.6±0.9 | 7.7±1.6 |
| **FOCUS** |  |  |  |  |
| DWI_b0_ | 21.3±6.8 | 14.6±9.5 | 16.2±5.0 | 24.9±4.3 |
| DWI_b10_ | 24.3±9.7 | 15.4±9.3 | 19.6±5.5 | 31.3±7.0 |
| DWI_b30_ | 19.3±6.6 | 13.9±6.6 | 18.7±5.2 | 29.6±7.6 |
| DWI_b60_ | 20.4±5.7 | 12.6±5.7 | 18.8±4.9 | 28.0±6.0 |
| DWI_b90_ | 19.7±12.1 | 11.7±4.3 | 18.6±3.8 | 28.2±8.5 |
| DWI_b150_ | 15.5±6.8 | 10.7±2.9 | 19.2±3.3 | 27.0±4.8 |
| DWI_b250_ | 11.0±4.0 | 9.3±2.5 | 19.4±3.1 | 26.2±4.6 |
| DWI_b500_ | 13.2±8.6 | 9.0±2.6 | 23.3±3.7 | 28.2±3.7 |
| DWI_b700_ | 10.1±2.6 | 8.4±1.9 | 22.1±4.0 | 27.0±4.8 |

Figure S7. Representative subject-specific simulated IVIM curves from the same participant with usable MUSE and FOCUS data, illustrating perfusion-related signal behavior in the PSD, CSF, GM, and WM derived from FOCUS data. (a) Semi-logarithmic plots of the simulated IVIM signal (C_IVIM_) and diffusion line (D_line_). (b) Corresponding normalized curves (norm_C_IVIM_ and norm_ D_line_). (c) Percentage difference (pseudo-diffusion residual) between norm_C_IVIM_ and norm_ D_line_ as a function of b-value. In each row, the left panels show the full b-value range, whereas the right panels provide an enlarged view of the low b-value range highlighted by the dashed boxes. Vertical dotted lines in (a) show the b-value at which the separation between C_IVIM_ and D_line_ becomes visually apparent, corresponding to the 0.2% pseudo-diffusion residual threshold indicated by the horizontal dotted line in (c). The pseudo-diffusion residual quantifies perfusion-related signal contribution and identifies the transition toward diffusion-dominated signal decay.
